# Utility and Wearability of the hitoe^®^ Wearable ECG Monitoring System II for Detecting Covert Paroxysmal Atrial Fibrillation in Patients with Suspected ESUS Across Inpatient and Outpatient Settings: ACROSS-AF in ESUS

**DOI:** 10.3390/neurolint18070134

**Published:** 2026-07-13

**Authors:** Hisanao Akiyama, Yasutaka Watanabe, Takayuki Fukano, Takahiro Shimizu, Yoshihisa Yamano

**Affiliations:** Department of Neurology, St. Marianna University School of Medicine, 2-16-1 Sugao, Miyamae-ku, Kawasaki 216-8511, Kanagawa, Japan; yasutaka.watanabe@marianna-u.ac.jp (Y.W.); t.fukano@marianna-u.ac.jp (T.F.); shimi-taka@marianna-u.ac.jp (T.S.); yyamano@marianna-u.ac.jp (Y.Y.)

**Keywords:** embolic stroke of undetermined source, covert paroxysmal atrial fibrillation, garment-type wearable ECG monitoring

## Abstract

**Background/Objectives**: Detecting covert paroxysmal atrial fibrillation (AF) in patients with suspected embolic stroke of undetermined source (ESUS) is important for secondary prevention. This study evaluated the feasibility, AF detection, and wearability of the hitoe^®^ wearable electrocardiogram (ECG) monitoring system II, a Holter-type device recording continuously for up to 14 days. **Methods**: Between March 2022 and October 2023, 31 patients with suspected ESUS were enrolled. After excluding two cases, 29 patients (mean age 74.7 ± 17.3 years; 16 men) underwent ECG monitoring. Clinical outcomes were analyzed in 27 acute-phase patients and wearability in 24 questionnaire respondents. ECG recordings and questionnaire responses were analyzed descriptively, with between-group comparisons. **Results**: In 29 monitored patients, the mean recording duration was 12.4 ± 3.7 days, the ECG acquisition rate was 64.0 ± 23.4% (median 72.1%), and the mean analyzable duration was 8.1 ± 3.7 days. In the 27 acute-phase patients, covert paroxysmal AF was detected in 2 patients (7.4%), on days 1, 3, and 15 in one patient and on day 7 in the other. In AF-positive patients, the mean ectopic burden was 1.33% for supraventricular and 0.24% for ventricular activity. Wearability was favorable: 77.8% reported no interference with daily activities, none reported sleep disturbance, and 72.7% adapted within 1–4 days. **Conclusions**: The hitoe^®^ wearable ECG monitoring system II enabled prolonged monitoring across inpatient and outpatient settings, detecting covert paroxysmal AF in 7.4% of acute-phase patients with suspected ESUS. These findings support garment-type wearable ECG monitoring as a non-invasive option for extended rhythm surveillance.

## 1. Introduction

Embolic stroke of undetermined source (ESUS) represents a subgroup of non-lacunar ischemic stroke in which no definite embolic source can be identified despite standardized diagnostic evaluation. Among the potential etiologies—including low-risk cardiac sources, arterial embolism, paradoxical embolism, and malignancy-related embolism—covert paroxysmal atrial fibrillation (AF) is considered one of the most frequent and clinically important causes. Detection of AF is essential for selecting appropriate secondary prevention strategies, particularly initiation of direct oral anticoagulants.

Short-duration monitoring by conventional 24 h Holter electrocardiogram (ECG) monitoring is routinely performed in clinical practice in Japan; however, its sensitivity for detecting covert paroxysmal AF remains limited. Numerous studies have shown that extending the monitoring duration markedly increases the AF detection rate, and a variety of long-term monitoring devices, including patch-type, garment-type, handheld, smartphone-based, smartwatch-based, and implantable loop recorders, are now available for clinical use. Despite this expansion of monitoring options, the choice of device is often driven by provider preference or workflow rather than patient-centered considerations, with evaluations of device usability from the patient perspective remaining scarce. The hitoe^®^ wearable ECG monitoring system II (hitoe^®^ system II) is a wearable, Holter-type device used in clinical cardiology practice. It allows patients to independently wear and remove the device, imposes minimal physical burden, and enables continuous ECG recording for up to 14 days without professional assistance. Although its feasibility and clinical applicability have been reported in cardiology settings, evidence regarding its application in the stroke field, particularly for detection of covert paroxysmal AF in ESUS, remains limited. Furthermore, its wearability in this population has not been systematically assessed.

In this study, we evaluated (1) the ability of the hitoe^®^ system II to detect covert paroxysmal AF in patients with ESUS and (2) the wearability of this device based on patient-reported outcomes. The aim of the study, known as ACROSS-AF in ESUS (Atrial Cardiac Rhythm Observation Spanning Inpatient to Outpatient Care Using Wearable Dry-Electrode “hitoe^®^” in ESUS), was to provide patient-centered evidence supporting long-term ECG monitoring from the acute inpatient phase through outpatient follow-up.

## 2. Materials and Methods

### 2.1. Study Population

**Figure 1:**
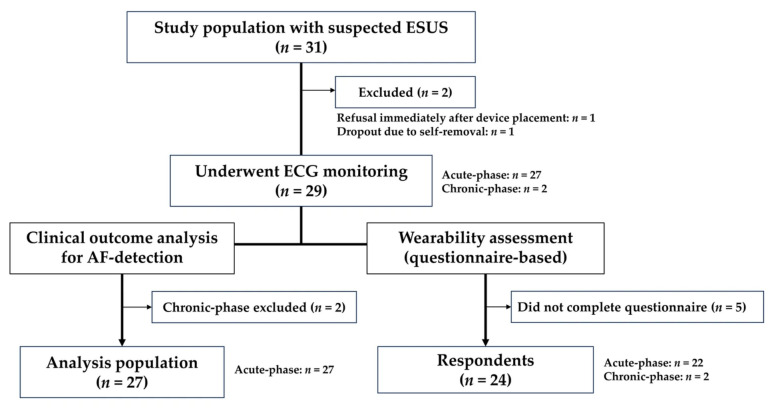
Study population. A total of 31 patients were enrolled, of whom two were excluded from monitoring after device placement. The remaining 29 patients completed monitoring, including 27 acute-phase patients and 2 chronic-phase patients. The clinical outcome analysis for AF detection included 27 acute-phase patients, and the wearability analysis included 24 patients, comprising acute- and chronic-phase patients who completed the questionnaire.

We prospectively enrolled 31 patients (17 men; mean age 74.4 ± 17.1 years) who were admitted to our hospital within 7 days of the onset of ischemic stroke between March 2022 and October 2023. Patients were eligible for inclusion if an embolic mechanism was suspected and no AF had been detected by any ECG monitoring modality for at least 24 h before enrollment, resulting in a diagnosis of suspected ESUS. Because the extent of embolic-source evaluation was limited in some patients during the COVID-19 pandemic, the study population is described as patients with suspected ESUS rather than uniformly confirmed ESUS. Among the 31 enrolled patients, two were excluded from monitoring after device placement, leaving 29 patients who completed monitoring: 27 underwent acute-phase monitoring, and 2 underwent chronic-phase monitoring (Figure 1). Participants wore the hitoe^®^ system II continuously for up to 14 days, except during bathing or magnetic resonance imaging procedures. ECG data were anonymized and centrally reviewed for AF detection. AF was defined as an irregularly irregular rhythm without P waves lasting ≥30 s. We evaluated the frequency, timing, and duration of AF episodes, as well as clinical characteristics, including age, sex, comorbidities, medical history, CHADS_2_ and CHA_2_DS_2_-VASc scores, serum N-terminal pro-B-type natriuretic peptide (NT-proBNP) and D-dimer levels, 24-h Holter findings, and carotid, transthoracic, cardiac, and lower-extremity venous ultrasonography, and assessed their associations with AF detection.

After the monitoring period, patients completed a structured questionnaire assessing wearability, covering domains including ease of use, comfort, and impact on daily activities with specific items such as fabric comfort, tightness, device detachment, itching or rash, interference with clothing, sleep disturbance, and time required to adapt to the hitoe^®^ system II. The wearability questionnaire was developed specifically for this exploratory feasibility study to assess patient-reported wearability and was not formally validated. The full questionnaire is provided in the Supplementary Data. The study was approved by the St. Marianna University School of Medicine Bioethics Committee (approval number 5563). All patients who participated in the study provided informed consent.

### 2.2. Participants: Inclusion and Exclusion Criteria

Inclusion criteria were as follows: (1) acute ischemic stroke within 7 days of onset for the clinical outcome analysis or patients in either the acute or chronic phase for the wearability analysis; (2) suspected ESUS with no AF detected on any ECG monitoring modality, including Holter ECG monitoring, during at least 24 h of rhythm monitoring before enrollment; (3) age ≥ 20 years at the time of consent; and (4) written informed consent obtained from the patient or a legally authorized representative.

Exclusion criteria were as follows: (1) pregnancy or possible pregnancy; (2) inability to wear ECG electrodes owing to dermatologic conditions; (3) known allergy to glycerin solution or skin adhesives; (4) any condition judged by the attending physician to make participation inappropriate.

### 2.3. hitoe^®^ System II

**Figure 2:**
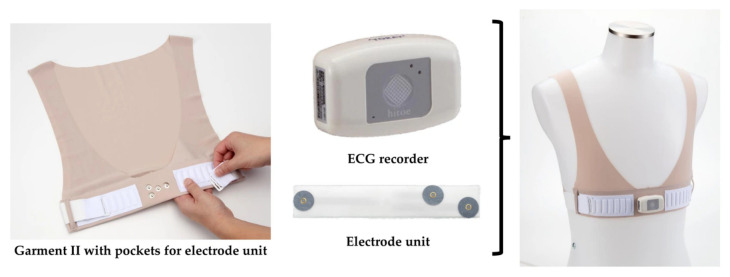
Components of the hitoe^®^ system II. The system consists of three components: a garment with pockets for the electrode unit, a long-term ECG recorder, and an electrode unit.

The hitoe^®^ system II (Figure 2) consists of three components: the hitoe^®^ garment II for use with the hitoe^®^ electrode unit (Sunrich Mode Co., Ltd., Tokyo, Japan), an EV-301 long-term ECG recorder (Parama-Tech Co., Ltd., Fukuoka, Japan; certification number 230AFBZX00014000), and a hitoe^®^ electrode unit (Toray Medical Co., Ltd., Tokyo, Japan; registration number 13B1X00015000036). The hitoe^®^ electrode unit consists of dry electrodes, lead wires, snap buttons, and a bottle of glycerin solution supplied as part of the unit. A small amount of the supplied glycerin solution was applied to the electrode surface before use. The EV-301 recorder is waterproof (IPX2), reusable, and capable of single-channel ECG recording for up to 14 days. It measures 58.5 × 38.5 × 17.5 mm (width × depth × height), weighs approximately 31 g, and contains an integrated lithium-ion polymer battery.

**Figure 3:**
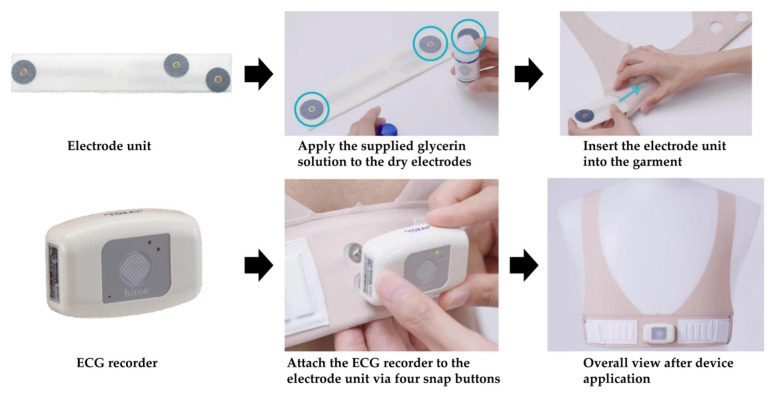
Application of the hitoe^®^ system II. For device setup, the supplied glycerin solution is applied to the dry electrodes, the electrode unit is inserted into the garment, and the ECG recorder is attached to the electrode unit via four snap buttons. Blue circles indicate electrode positions, and blue arrows indicate the insertion direction of the electrode unit.

For device setup (Figure 3), the electrode unit is inserted into the inner pocket of the garment so that the dry electrodes protrude through three openings. Patients then wear the garment, and the ECG recorder is attached to the four snap buttons located on the lower anterior chest area.

### 2.4. Statistical Analysis

The ECG data and responses to the questionnaire were analyzed descriptively. The clinical outcome analysis included 27 acute-phase patients, and the questionnaire-based wearability assessment included 24 patients, including chronic-phase patients (Figure 1). Variables analyzed included recording duration, ECG acquisition rate, number of analyzable days, AF detection timing, and AF episode duration. The ECG acquisition rate was calculated using the total recording time during the monitoring period as the denominator, including temporary interruptions related to patients’ daily activities (e.g., bathing), and the analyzable recording time after excluding segments affected by noise or artifacts as the numerator. In this analysis, non-wear periods and signal artifacts were not explicitly distinguished and were collectively treated as non-analyzable segments, in accordance with the predefined ECG reporting workflow used in this study.

Continuous variables were compared between groups using the Mann–Whitney *U* test. Differences across usage settings (inpatient, inpatient-to-outpatient transition, outpatient) were compared between groups using the Kruskal–Wallis test. Categorical variables were compared using the chi-squared test or Fisher’s exact test, depending on sample size. Statistical analyses were performed using custom scripts written in Python (Python Software Foundation, Wilmington, DE, USA; version 3.12.10, 64-bit) with the SciPy library version 1.17.1 (SciPy Developers), using standard implementations in scipy.stats. The analysis code is available from the corresponding author upon reasonable request. Given the small number of AF-positive cases, analyses involving AF-positive patients were primarily descriptive and exploratory by design. A *p*-value of <0.05 was considered statistically significant.

Primary endpoints were the AF detection rate and the timing and duration of AF episodes during recording with the hitoe^®^ system II. Secondary endpoints included associations between AF detection and CHADS_2_ and CHA_2_DS_2_-VASc scores, serum NT-proBNP and D-dimer levels, presence of patent foramen ovale or lower-extremity venous thrombosis, and wearability outcomes.

## 3. Results

### 3.1. Completion of Monitoring

Of the 31 enrolled patients with suspected ESUS, two were excluded (one for declining to continue immediately after device placement and the other for removing the device independently), resulting in 29 patients (16 men; mean age 74.7 ± 17.3 years) who completed monitoring using the hitoe^®^ garment II. Among these, 27 acute-phase patients were included in the clinical outcome analysis. For the wearability assessment, five patients who did not complete the questionnaire were excluded, resulting in 24 patients who completed the questionnaire (Figure 1).

During the COVID-19 pandemic, a complete embolic-source evaluation could not be performed in all patients. Among those who underwent transthoracic and transesophageal echocardiography, no patent foramen ovale was identified. Lower-extremity venous ultrasonography detected venous thrombosis in five patients (one in the AF-positive group and four in the AF-negative group) (Table 1).

### 3.2. Utility of the hitoe^®^ Wearable ECG Monitoring System II in the Acute-Phase Cohort

#### 3.2.1. Detection of Covert Paroxysmal AF

**Figure 4:**
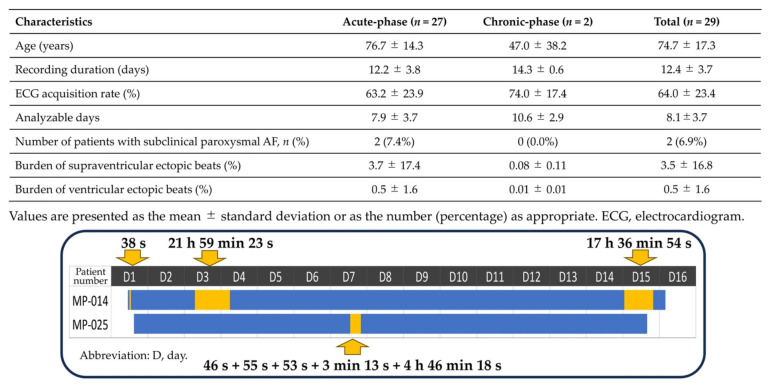
Detection of covert paroxysmal AF. Covert paroxysmal AF was detected in 2 (7.4%) of 27 patients (on days 1, 3, and 15 in one patient and on day 7 in the other). Blue bars indicate recording periods, orange bars indicate AF episodes, and adjacent numbers indicate episode duration. Yellow arrows indicate the timing of AF detection. The cumulative AF duration was 39 h 36 min 55 s in MP-014 and 4 h 52 min 05 s in MP-025. AF, atrial fibrillation.

The 27 acute-phase patients had a mean age of 76.7 ± 14.3 years (median 79.0). The mean monitoring duration was 12.2 ± 3.8 days (median 14.0), the mean ECG acquisition rate was 63.2 ± 23.9% (median 72.1%), and the mean number of analyzable days was 7.9 ± 3.7 (median 8.4) (Table 1). Covert paroxysmal AF was detected in 2 of 27 patients (7.4%). AF was detected on days 1, 3 (single episodes on each day), and 15 (three episodes) in one patient and on day 7 (five episodes) in the other (Figure 4). In MP-014, AF episodes lasted 38 s on day 1, 21 h 59 min 23 s on day 3, and 17 h 36 min 54 s on day 15; the cumulative AF duration was 39 h 36 min 55 s, and the longest AF episode was 21 h 59 min 23 s. In MP-025, five AF episodes were detected on day 7, lasting 46 s, 55 s, 53 s, 3 min 13 s, and 4 h 46 min 18 s; the cumulative AF duration was 4 h 52 min 5 s, and the longest AF episode was 4 h 46 min 18 s.

In both AF-positive patients, apixaban had already been initiated before application of the hitoe^®^ system II, at 3 days after stroke onset in MP-014 and 14 days after stroke onset in MP-025. Therefore, AF episodes recorded during hitoe^®^ monitoring did not trigger initiation of anticoagulation. Rather, the hitoe^®^ system II provided additional rhythm documentation of paroxysmal AF during prolonged monitoring. In MP-014, AF episodes were documented during both hospitalization and post-discharge follow-up, illustrating the feasibility of continuous rhythm surveillance across the transition from inpatient to outpatient care. No bleeding events or recurrent ischemic events were observed during hospitalization in these two patients.

Because only two AF-positive cases were identified, no formal statistical comparison was performed for ectopic burden in the AF-positive subgroup. The observed ectopic burden in these cases was therefore interpreted descriptively, with supraventricular ectopy appearing more prevalent than ventricular ectopy.

**Figure 5:**
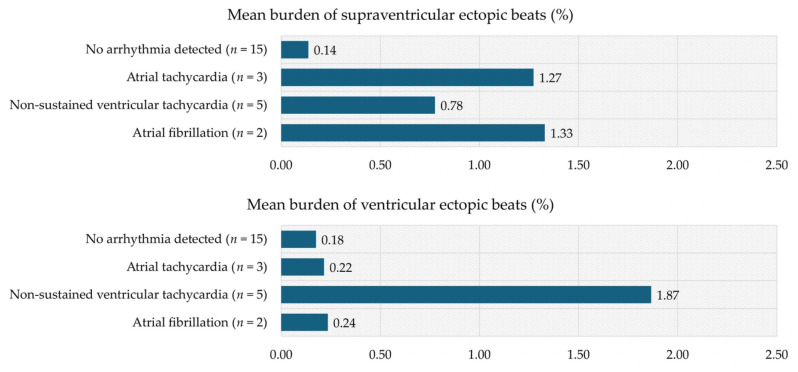
Descriptive distribution of supraventricular and ventricular ectopy in AF-positive patients. Because only two AF-positive cases were identified, no formal statistical comparison was performed.

Examination of the relationship between covert paroxysmal AF and the burden of supraventricular and ventricular ectopy suggested a higher rate of supraventricular ectopic beats than ventricular ectopic beats in AF-positive patients (1.33% vs. 0.24%) (Figure 5).

#### 3.2.2. Patient Characteristics by AF Detection Status

There were no significant differences in age, sex, comorbidities, CHADS_2_ and CHA_2_DS_2_-VASc scores, NT-proBNP and D-dimer levels, or ultrasound findings, likely because of the small number of cases in the AF-positive group (Table 1).

### 3.3. Wearability of the hitoe^®^ System II in Acute-Phase and Chronic-Phase Cohorts

#### 3.3.1. Ease of Wearing and Removing the Garment

**Figure 6:**
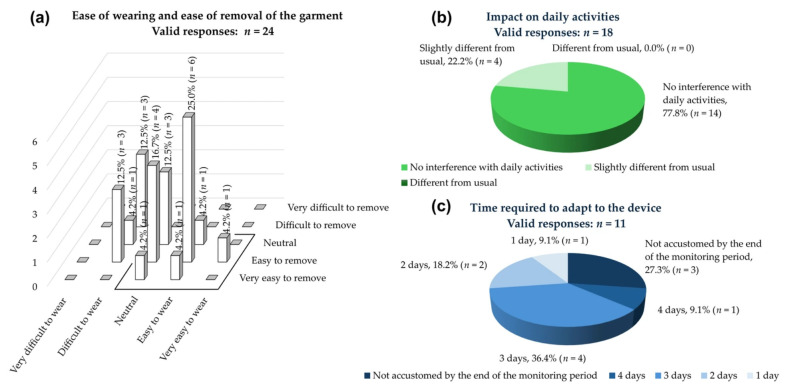
Responses to the wearability questionnaire: (**a**) Wearability was evaluated by visualizing the distribution of responses for ease of wearing and ease of removal using a three-dimensional bar chart. The framed area indicates acceptable responses, defined as “very easy,” “easy,” or “neutral” for both wearing and removal; 17 of 24 patients (70.8%) were included in this area. (**b**) Fourteen of 18 patients (77.8%) reported no interference with daily activities. In panel (**b**), the deep green response category had no selections (0.0%, *n* = 0) and is therefore not visible as a bar. (**c**) Eight of 11 patients (72.7%) adapted to the device within 1–4 days. Colors are used only to distinguish response categories.

Among the 24 respondents, 17 patients (70.8%) rated the garment as “very easy,” “easy,” or “neutral” to put on. Similarly, 21 patients (87.5%) rated removal of the garment as “very easy,” “easy,” or “neutral.” When the distributions of responses for ease of wearing and ease of removal were visualized using a three-dimensional bar chart, cases corresponding to “very easy,” “easy,” or “neutral” for both wearing and removal accounted for 17 of 24 patients (70.8%) (Figure 6a). There were few cases outside these categories.

#### 3.3.2. Fabric Comfort

Among 20 respondents, 14 (70.0%) rated the fabric as “good” or “very good”.

#### 3.3.3. Tightness of the Garment

Among 19 respondents, 13 (68.4%) rated the tightness as “just right”.

#### 3.3.4. Impact on Daily Activities

Among 18 respondents, 14 (77.8%) reported no interference with daily activities (Figure 6b).

#### 3.3.5. Time Required to Adapt

Among 11 respondents, 8 (72.7%) adapted to the device within 1–4 days (Figure 6c).

#### 3.3.6. Detachment of the Device

Among 24 respondents, 21 (87.5%) reported no instances of device detachment.

#### 3.3.7. Impact on Clothing

Among 18 respondents, only 1 patient (5.6%) reported concern about the bulging of the device, while 12 (66.7%) reported no concern.

#### 3.3.8. Impact on Sleep

Among 18 respondents, no patient reported sleep disturbance, and 8 (44.5%) reported sleeping as usual.

### 3.4. hitoe^®^ System II Usage Settings in Acute-Phase and Chronic-Phase Cohorts

Differences according to three usage settings (inpatient-only [*n* = 17], inpatient-to-outpatient transition [*n* = 9], and outpatient-only [*n* = 3]) were examined in the 27 acute-phase and 2 chronic-phase patients (Table 2). Several variables differed significantly across usage settings; however, these exploratory comparisons should be interpreted cautiously because phase and patient background were closely linked to usage setting. In the inpatient-to-outpatient transition group, the mean number of inpatient monitoring days was 6.7 ± 3.2, and the mean number of outpatient monitoring days was 8.1 ± 2.8. AF detection by usage setting was as follows: 0/3 (0%) in the outpatient-only group, 1/17 (5.9%) in the inpatient-only group, and 1/9 (11.1%) in the inpatient-to-outpatient transition group. In the transition group, ECG monitoring was maintained across both inpatient and outpatient phases in selected patients.

## 4. Discussion

Multiple potential embolic sources have been proposed for ESUS, including low-risk cardiac sources (such as myxomatous mitral valve disease with prolapse, mitral annular calcification, aortic stenosis, calcified aortic valves, atrial tachyarrhythmias, and atrial septal aneurysm), covert paroxysmal AF, nonbacterial thrombotic endocarditis, tumor embolism associated with occult malignancy, arterial embolism (aortic arch plaques and non-stenotic ulcerated intracranial arterial plaques), and paradoxical embolism (patent foramen ovale, atrial septal aneurysm, and pulmonary arteriovenous fistula). Covert paroxysmal AF is considered to be the most frequent and clinically important etiology. Current stroke guidelines recommend at least 24 h of ECG monitoring to detect AF; however, the optimal monitoring duration and type of device remain undefined. Therefore, monitoring practices vary widely across institutions, and covert paroxysmal AF may be missed. Moreover, 20–40% of ischemic strokes remain classified as ESUS, even after extensive evaluation. Short-duration Holter monitoring alone tends to underestimate covert paroxysmal AF, and observational studies consistently show that longer monitoring durations increase AF detection rates.

The CRYSTAL AF trial reported AF detection rates of 8.9% at 6 months and 12.4% at 12 months using implantable cardiac monitors, significantly outperforming conventional follow-up [1]. In the EMBRACE trial, Gladstone et al. found that 30-day event monitoring detected AF lasting ≥30 s in 16.1% of patients (≥2.5 min in 9.9%), whereas 24 h Holter monitoring detected 3.2% (≥2.5 min in 2.5%) and was associated with a higher rate of anticoagulation initiation (18.6% vs. 11.1%) [2]. Furthermore, Šaňák et al. reported an AF detection rate of 10.2% using a 3-week monitor in younger stroke patients [3]. Across these studies, AF detection rates generally range from 6% to 16%, depending on the duration of monitoring. The AF detection rate of 7.4% in our study falls within this expected range for a 14-day garment-type monitor, being comparable with the findings of the EDUCATE-ESUS trial (5.8% over 7 days) [4] and the Crypto-AF study (9.9% over 28 days) [5]. These findings support the rationale for prolonged rhythm monitoring in selected patients with ESUS, but direct comparisons with prior studies should be interpreted cautiously because monitoring duration, device type, patient selection, and definitions of analyzable data differed across studies.

In clinical practice, patients with non-lacunar stroke who show no AF on 24-h Holter monitoring are often diagnosed with ESUS and started on antiplatelet therapy according to guideline recommendations. While not incorrect, this approach risks missing covert paroxysmal AF. Long-term ECG monitoring provides an opportunity to detect covert paroxysmal AF that would otherwise remain unrecognized, allowing reclassification from ESUS to cardioembolic stroke and informing individualized secondary-prevention strategies. Management after AF detection should be individualized according to thromboembolic risk, bleeding risk, comorbidities, and overall clinical context and may include oral anticoagulation or other risk-based management approaches [6,7].

A variety of monitoring devices are now available worldwide, including conventional 24 h Holter monitors, patch-type monitors, garment-type monitors, handheld ECG devices, smartphone-based ECGs, smartwatch ECGs, broader multiparametric wearable systems, and implantable loop recorders. Except for the 24 h Holter, most allow extended monitoring and are useful for detecting covert paroxysmal AF that short-term monitoring may miss. However, device selection is often driven by provider or institutional preference rather than patient-centered evaluation, given that few studies have assessed usability from the patient perspective.

The hitoe^®^ system II has demonstrated utility in cardiology settings [8], but evidence in the stroke field has been limited, particularly for ESUS. In this study, we evaluated the clinical positioning of this garment-type wearable ECG monitoring system, focusing on AF detection, associations with other diagnostic findings, and patient-reported wearability, without making direct comparative claims against standard 24 h Holter monitoring or implantable monitoring devices. Accordingly, the hitoe^®^ system II should be viewed as a non-invasive, removable garment-type wearable ECG monitoring option that may be useful when prolonged but non-implantable rhythm surveillance is desired, particularly across inpatient and outpatient stroke-care settings. Its potential value lies not in replacing conventional Holter monitoring, patch monitors, or implantable loop recorders, but in offering an additional patient-centered monitoring option with distinct practical characteristics, including dry electrodes, clothing-like wearability, and flexible removal during hygiene care or medical procedures.

In our cohort, the mean interval between stroke onset and diagnosis was 1.5 ± 3.0 days, and the mean interval between diagnosis and device placement was 7.5 ± 8.3 days (median 6.0). In the stroke care setting, the hitoe^®^ system II enabled prolonged ECG monitoring for up to 14 days and achieved a mean ECG acquisition rate of 63.2 ± 23.9% (median 72.1%) on a mean of 7.9 ± 3.7 analyzable days.

The ECG acquisition rate may appear relatively modest in a real-world context. However, this value should be interpreted in the context of the recording methodology. The denominator included the entire monitoring period, including non-wear and other non-analyzable segments. Therefore, the reported acquisition rate represents a composite real-world metric rather than a direct measure of ECG signal quality during active wear and should be regarded as a conservative estimate.

Visual review of individual recordings showed substantial inter-patient heterogeneity, with some patients achieving consistently high acquisition rates and others exhibiting prolonged periods of non-analyzable data. In several cases, these periods were suggestive of intermittent non-wear or temporary device removal during hygiene care, medical procedures, or other daily activities. Consequently, the overall acquisition rate may underestimate effective ECG signal quality during active wear and may reduce sensitivity for detecting short or infrequent AF episodes.

The hitoe^®^ system II enables long-term monitoring for up to 14 days and can be worn like clothing using non-adhesive dry electrodes. This design may be particularly advantageous in stroke care settings, where multiple nursing and medical interventions are required during hospitalization.

The lower ECG acquisition rate observed in the inpatient group should be interpreted cautiously because patient characteristics and care-related factors may also have contributed. Because the number of patients in each subgroup was small, comparisons among monitoring settings should be regarded as exploratory and hypothesis-generating. Future studies should prospectively distinguish actual wear time from signal-quality loss by separately classifying non-wear, poor skin-electrode contact, motion artifacts, technical interruption, and other sources of non-analyzable data. Such analyses would allow a clearer distinction between patient adherence, care-related temporary removal, and device or signal performance and would help estimate the effect of non-analyzable periods on AF detection sensitivity.

Older adults with stroke often have cognitive or functional impairments, making device tolerability essential. The patients in our study, who were aged 73–87 years (median 78), wore the device for up to 14 days, with a median of 14.0 recording days, 8.6 analyzable days, and an ECG acquisition rate of 72.1%. These findings support the feasibility of prolonged garment-type ECG monitoring in selected older patients.

Our questionnaire revealed favorable wearability: approximately 70% rated the hitoe^®^ garment II as easy to wear/remove, comfortable in texture, and appropriate in tightness; nearly 90% reported no device detachment; and 70% adapted within 1–4 days. No patient reported sleep disturbance. Some patients reported concerns about device bulging or visibility under thin clothing, suggesting areas for improvement. Mild itching or rash occurred in eight patients but did not require treatment. Younger age was associated with higher rates of skin irritation, although there was no significant difference in the CHADS_2_ score, recording duration, or acquisition rate. Increased activity levels may contribute to irritation, and strategies such as garment changes after sweating or improved fitting instructions may help.

Although venous thrombosis was detected in several patients, these cases were considered to represent organized peripheral thrombi and were unlikely to be clinically relevant embolic sources. In addition, the extent of embolic-source evaluation was incomplete in some patients, which may have affected the diagnostic certainty of ESUS classification.

This study has some limitations. First, it was conducted during the COVID-19 pandemic, which complicated the process of obtaining consent from patients or their surrogates. Second, it was a single-center prospective study with a small sample size, which limits the generalizability of its findings. Importantly, only two AF-positive cases were identified, substantially limiting statistical power and precluding definitive conclusions regarding predictors of AF detection or associations with ectopic burden. Third, pandemic-related restrictions delayed some diagnostic evaluations, potentially postponing device placement. Finally, although the ECG acquisition rate was influenced by both non-wear and signal quality, these factors were not systematically quantified, and their relative contributions to non-analyzable ECG segments could not be distinguished. Consequently, the possibility that short or infrequent AF episodes were missed during non-analyzable periods cannot be excluded.

Because the observed AF detection rate was based on only two events, it should not be interpreted as a precise estimate for the broader suspected ESUS population. The wearability questionnaire was developed for this exploratory feasibility study and was not formally validated; moreover, missing questionnaire responses and exclusion of patients unable or unwilling to wear the device may have introduced selection and response bias. Finally, because embolic-source evaluation was incomplete in some patients, the diagnostic certainty of ESUS classification may have varied across the cohort.

## 5. Conclusions

In this evaluation of the hitoe^®^ system II in patients with suspected ESUS, prolonged garment-type ECG monitoring enabled rhythm surveillance across inpatient and outpatient settings and identified covert paroxysmal AF in 2 of 27 acute-phase patients. Wearability was generally favorable among questionnaire respondents. These findings support further investigation of garment-type wearable ECG monitoring as a non-invasive option for prolonged rhythm surveillance in patients with suspected ESUS.

## Figures and Tables

**Table 1:** Baseline characteristics and recording performance of the hitoe^®^ wearable ECG system II in 27 acute-phase patients with ESUS.

Characteristic	AF-Positive Group (*n* = 2)	AF-Negative Group (*n* = 25)	All (*n* = 27)
Age (years)	84.5 ± 7.8	76.1 ± 14.6	76.7 ± 14.3
Male sex	1	13	14
Comorbidities			
Hypertension	1	14	15
Diabetes mellitus	0	5	5
Dyslipidemia	0	11	11
Smoking history	0	9	9
CHADS_2_ score	2.0 ± 1.4	2.3 ± 1.4	2.3 ± 1.4
CHA_2_DS_2_-VASc score	3.5 ± 0.7	3.7 ± 1.8	3.7 ± 1.7
NT-proBNP level (pg/mL)	499.5 ± 368.4	2973.7 ± 7130.1	2783.4 ± 6872.3
NT-proBNP level, median [IQR] (pg/mL)	499.5 [369.3–629.8]	548.5 [213.0–1780.5]	548.5 [214.8–1555.0]
D-dimer level (ng/mL)	4.3 ± 0.1	2.1 ± 1.6	2.2 ± 1.6
D-dimer level, median [IQR] (ng/mL)	4.3 [4.2–4.3]	1.8 [0.8–2.6]	2.0 [0.9–2.7]
Interval from stroke onset to device application (days)	3.5 ± 0.7	10.0 ± 9.1	9.5 ± 8.9
Mean recording duration (days)	14.4 ± 0.5	12.1 ± 3.9	12.2 ± 3.8
Mean ECG acquisition rate (%)	63.5 ± 12.2	63.2 ± 24.7	63.2 ± 23.9
Mean analyzable days	9.1 ± 2.1	7.8 ± 3.8	7.9 ± 3.7
Ultrasonography			
Transthoracic echocardiography	2	25	27
Transesophageal echocardiography	0	5	5
Positive patent foramen ovale	0	0	0
Lower-extremity venous ultrasonography	2	19	21
Peripheral venous thrombosis	1	4	5

Data are shown as the mean ± standard deviation, median [interquartile range], or numbers, as appropriate. Median [interquartile range] values are additionally shown for NT-proBNP and D-dimer, and CHA_2_DS_2_-VASc scores are included as a reference measure. Baseline characteristics of 27 acute-phase patients with ESUS are shown. Patients were categorized into AF-positive (*n* = 2) and AF-negative (*n* = 25) groups; no significant differences were observed because of the small number of AF-positive cases. Hypertension was defined as a history or current diagnosis of hypertension. Diabetes mellitus was defined as a history of or current diagnosis of diabetes mellitus, including type 2 diabetes mellitus. Dyslipidemia was defined as a history or current diagnosis of dyslipidemia. Smoking history included both former and current smokers. AF, atrial fibrillation; ECG, electrocardiogram; ESUS, embolic stroke of undetermined source; NT-proBNP, N-terminal pro-B-type natriuretic peptide.

**Table 2:** Usage settings of the hitoe^®^ system II.

Variable	Overall	Inpatient	Inpatient-to-Outpatient Transition	Outpatient	*p*-Value	Statistical Analysis
Phase, *n* (%)	29 (100.0%)	17 (58.6%)	9 (31.0%)	3 (10.3%)		
Acute-phase, *n* (%)	27 (93.1%)	17 (100.0%)	9 (100.0%)	1 (33.3%)		
Chronic-phase, *n* (%)	2 (6.9%)	0 (0.0%)	0 (0.0%)	2 (66.7%)		
Male sex, *n* (%)	16 (55.2%)	7 (41.2%)	6 (66.7%)	3 (100.0%)		
ECG acquisition rate (%)	64.0 ± 23.4	54.2 ± 25.0	80.9 ± 8.6	68.1 ± 16.0	0.026	Kruskal–Wallis test
AF detected, *n* (%)	2 (6.9%)	1 (5.9%)	1 (11.1%)	0 (0.0%)		
Age (years)	74.7 ± 17.3	82.2 ± 11.0	67.7 ± 15.6	52.7 ± 28.7	0.006	Kruskal–Wallis test
CHADS_2_ score	2.2 ± 1.4	2.8 ± 1.4	1.3 ± 1.0	1.7 ± 0.6	0.02	Kruskal–Wallis test
CHA_2_DS_2_-VASc score	3.6 ± 1.7	4.5 ± 1.3	2.3 ± 1.5	2.0 ± 0.0	<0.001	Kruskal–Wallis test
NT-proBNP level (pg/mL)	2686 ± 6758	4137 ± 8255	237 ± 213	50	0.003	Kruskal–Wallis test
NT-proBNP level, median [IQR] (pg/mL)	548 [212–1409]	1082 [425–2080]	210 [72–239]	50 [50–50]	0.003	Kruskal–Wallis test
D-dimer level (ng/mL)	2.1 ± 1.6	2.6 ± 1.7	1.3 ± 1.3	0.5	0.033	Kruskal–Wallis test
D-dimer level, median [IQR] (ng/mL)	1.8 [0.7–2.7]	2.3 [1.4–3.2]	0.7 [0.3–1.8]	0.5 [0.5–0.5]	0.033	Kruskal–Wallis test
Interval between stroke onset and consent	1.4 ± 3.0	1.3 ± 3.1	1.9 ± 3.2	0.7 ± 1.2	0.731	Kruskal–Wallis test
Interval between diagnosis and consent	16.6 ± 48.6	7.5 ± 10.1	7.0 ± 3.6	137 ± 175	0.058	Kruskal–Wallis test
Interval between consent and study device application	0.4 ± 1.1	0.6 ± 1.3	0.2 ± 0.4	0.0 ± 0.0	0.739	Kruskal–Wallis test
Monitoring duration (days)	13.4 ± 3.8	12.4 ± 4.6	14.8 ± 1.1	15.3 ± 0.6	0.217	Kruskal–Wallis test
Inpatient monitoring period (days)	9.3 ± 5.7	12.4 ± 4.6	6.7 ± 3.2	0.0 ± 0.0	0.001	Kruskal–Wallis test
Outpatient monitoring period (days)	4.1 ± 5.6	0.0 ± 0.0	8.1 ± 2.8	15.3 ± 0.6	<0.001	Kruskal–Wallis test
Recording duration (days)	12.4 ± 3.7	11.4 ± 4.6	13.7 ± 1.2	14.1 ± 0.6	0.356	Kruskal–Wallis test
Analyzable days	8.1 ± 3.7	6.2 ± 3.6	11.1 ± 1.0	9.7 ± 2.7	0.002	Kruskal–Wallis test
Burden of ventricular premature beats (%)	0.5 ± 1.6	0.8 ± 2.0	0.05 ± 0.10	0.01 ± 0.01	0.008	Kruskal–Wallis test
Burden of supraventricular premature beats (%)	3.5 ± 16.8	5.9 ± 21.8	0.09 ± 0.15	0.09 ± 0.08	0.051	Kruskal–Wallis test

Data are shown as the mean ± standard deviation, median [interquartile range], or numbers (percentages) as appropriate. Median [interquartile range] values are additionally shown for NT-proBNP and D-dimer, and CHA_2_DS_2_-VASc scores are included as a reference measure. Several variables differed significantly across monitoring settings; however, these exploratory comparisons should be interpreted cautiously because phase and baseline characteristics differed among settings. Phase distribution was presented descriptively without formal statistical testing. The AF detection rate was 0% (0/3) in the outpatient group, 5.9% (1/17) in the inpatient group, and 11.1% (1/9) in the inpatient-to-outpatient transition group. AF, atrial fibrillation; ECG, electrocardiogram; NT-proBNP, N-terminal pro-B-type natriuretic peptide.

## Data Availability

The original contributions presented in this study are included in the article. Further inquiries can be directed to the corresponding author.

## Supplementary Materials

The following supporting information can be downloaded at: https://www.mdpi.com/article/10.3390/neurolint18070134/s1.

## Abbreviations

The following abbreviations are used in this manuscript:

AF	Atrial fibrillation
ECG	Electrocardiogram
ESUS	Embolic stroke of undetermined source
NT-proBNP	N-terminal pro-B-type natriuretic peptide
CHADS_2_	Congestive heart failure, hypertension, age ≥ 75 years, diabetes mellitus, and stroke/transient ischemic attack score
CHA_2_DS_2_-VASc	Congestive heart failure, hypertension, age ≥ 75 years, diabetes mellitus, stroke/transient ischemic attack, vascular disease, age 65–74 years, and sex category score
