# Supplementary material for: Utility and Wearability of the hitoe® Wearable ECG Monitoring System II for Detecting Covert Paroxysmal Atrial Fibrillation in Patients with Suspected ESUS Across Inpatient and Outpatient Settings: ACROSS-AF in ESUS
Source: Neurol Int. 2026 Jul 13;18(7):134. doi: 10.3390/neurolint18070134 (PMC13416157; doi:10.3390/neurolint18070134)
Supplement: Supplementary file 1 [file neurolint-18-00134-s001.zip › neurolint-4374851-supplementary.pdf]

Questionnaire for Patients

hitoe® Wearable ECG Monitoring System II Questionnaire (for Patients)

This questionnaire was created to understand the condition of patients who participated in this clinical study and to gather feedback on the hitoe® Wearable ECG Monitoring System II (hereafter referred to as the “wearable ECG system”) provided during the study.

Your responses will not affect your medical care in any way.

If you do not wish to participate in this survey, please check the box below.

☐ *I do not agree to participate in this survey.*

1. Ease of Wearing and Removal

(1) Please evaluate how easy it was to put on the wearable ECG system.

☐ Very easy

☐ Easy

☐ Neutral

☐ Difficult

☐ Very difficult

(2) Please evaluate how easy it was to remove the wearable ECG system.

☐ Very easy

☐ Easy

☐ Neutral

☐ Difficult

☐ Very difficult

(3) Were you able to put on and remove the system by yourself?

☐ Yes

☐ No (assistance from family members or caregivers, such as nurses, was required)

(4) If you have any comments regarding the use or handling of the garment (positive or negative), please describe them below.

(e.g., “Inserting the electrodes was cumbersome.”)

(5) Did the ECG recorder detach from the garment during use?

☐ Yes

☐ No

(6) Did the electrodes show edge lifting (partial detachment at the edges) or become bent during use?

☐ Yes

☐ No

(7) If detachment or electrode issues occurred, please describe when and how often they happened.

2. Comfort and Design

(1) How did you feel about the texture of the fabric?

☐ Very good

☐ Good

☐ Neutral

☐ Poor

☐ Very poor

(2) How did the tightness of the garment feel?

☐ Too tight

☐ Just right

☐ Too loose

(3) How did you feel about the two-week wearing period?

☐ Too short

☐ Appropriate

☐ Too long

(4) Did you experience itching or a skin rash at areas in contact with the garment?

☐ No

☐ Yes

If yes, please describe the symptoms.

(5) How did you feel about the design of the garment?

☐ Very good

☐ Good

☐ Neutral

☐ Poor

☐ Very poor

(6) Compared with other ECG examinations (e.g., conventional Holter monitoring), how comfortable was the wearable ECG system?

☐ Much more comfortable

☐ More comfortable

☐ About the same

☐ Less comfortable

☐ Much less comfortable

3. Impact on Daily Life

(1) Sleep: How did wearing the device affect your sleep?

☐ Slept as usual

☐ Slightly difficult to sleep

☐ Difficult to sleep

(2) Eating: How did wearing the device affect eating?

☐ No change

☐ Slightly difficult

☐ Difficult

(3) Clothing: How did wearing the device affect your clothing?

☐ Not noticeable

☐ Slightly noticeable bulge of the device

☐ Noticeable bulge of the device

(4) Other aspects of daily life

☐ No change

☐ Slight changes

☐ Many changes

If you selected "slight changes" or "many changes," please describe the reasons.

(5) If there was an impact on daily life, how long did it take you to get used to wearing the device?

☐ About ( ) days

☐ I did not get used to it during the wearing period

(6) How many times did you change the garment during the study period?

( ) times

(7) In what situations did you change the garment?

(e.g., after rehabilitation sessions)

4. Additional Comments

If you have any other comments, suggestions, or requests regarding your experience with the wearable ECG system, please feel free to write them below.

Section to be completed by healthcare professionals

Patient ID: ____________

Sex: ☐ Male ☐ Female

Garment size: ☐ S ☐ M ☐ L
